# Clinical Pilot Study of Rectal Suppository Containing Combined Extract of *Cissus quadrangularis* Linn. and *Acmella paniculata* (Wall ex. DC.) R. K. Jansen in Acute Hemorrhoids

**DOI:** 10.1155/2021/5605323

**Published:** 2021-11-02

**Authors:** Kanokporn Niwatananun, Wirat Niwatananun, Sirivipa Piyamongkol, Darunee Hongwiset, Chidchanok Ruengorn, Kiatkriangkrai Koyratkoson, Panee Sirisa-ard, Sunee Chansakaow, Songwut Yotsawimonwat, Chalermpong Saenjum, Ampai Phrutivorapongkul, Suporn Charumanee

**Affiliations:** ^1^Department of Pharmaceutical Care, Faculty of Pharmacy, Chiang Mai University, Chiang Mai 50200, Thailand; ^2^Department of Pharmaceutical Sciences, Faculty of Pharmacy, Chiang Mai University, Chiang Mai 50200, Thailand; ^3^Pharmacoepidemiology and Statistics Research Center (PESRC), Faculty of Pharmacy, Chiang Mai University, Chiang Mai 50200, Thailand; ^4^Cluster of Excellence on Biodiversity-based Economics and Society (B. BES-CMU), Chiang Mai University, Chiang Mai 50200, Thailand

## Abstract

**Background:**

*Cissus quadrangularis* Linn. (CQ) is a medicinal plant with good evidence for the treatment of hemorrhoids, listed in the Thai National List of Herbal Products in the oral dosage form. *Acmella paniculata* (Wall ex. DC.) R. K. Jansen. (AP) is a medicinal plant with a local anesthetic effect.

**Objective:**

To investigate the potential of rectal suppositories containing CQ and AP extracts to alleviate symptoms of hemorrhoids compared with the commercialized rectal suppository containing hydrocortisone and cinchocaine.

**Materials and Methods:**

Hemorrhoid outpatients (*n* = 105) with different severity grades (I, II, or III) from eight hospitals in northern Thailand were included in this study. Hemorrhoid severity was graded by proctoscopy associated with either anal pain or bleeding related to hemorrhoids or both. The patients were randomly allocated to two groups: CQ-AP group (*n* = 52) or the commercialized rectal suppository group (*n* = 53). One suppository was rectally administered twice daily in the morning and at bedtime for seven days. Evaluations were performed by physicians on days 1, 4, and 8 of the study. The primary endpoints were bleeding and prolapse size, while the secondary endpoint was anal pain.

**Results:**

Baseline demographics, lifestyle, constipation, number of prolapses, grade of hemorrhoid severity, and duration of experiencing hemorrhoids were comparable in both groups of patients. The effects of CQ-AP and the commercialized rectal suppository on bleeding, prolapse size, and anal pain were comparable. The patients in both groups were satisfied with both products at comparable levels and stated a preference for further use in the case of hemorrhoids recurrence. In terms of safety, the patients in the commercialized rectal suppository group experienced a higher incidence of adverse events, including anal pain and bleeding.

**Conclusion:**

Rectal suppositories containing a combined extract of CQ and AP show potential in alleviating hemorrhoidal symptoms with a good safety profile.

## 1. Introduction

Hemorrhoid is an anorectal disease caused by venous dilatation in the rectum and anus. Clinical manifestations of hemorrhoids commonly found in the early phase include bleeding during or after defecation. Pain, itching, and/or prolapse in the anus can sometimes be found [1]. The prevalence of hemorrhoids investigated in the monks living in Bangkok was 4% [2]. In South Korea, 14.4% of the adult population suffers from hemorrhoids-related symptoms [3]. At present, physicians usually prescribe conventional medications to alleviate hemorrhoidal symptoms. Following Thai folklore remedies, *Cissus quadrangularis* Linn. (CQ) has long been used for hemorrhoids because of its vasoconstrictive effect and anti-inflammatory activity [4, 5]. In addition, CQ also has an analgesic effect [6]. Regarding the safety profile, CQ does not cause serious adverse drug events [7, 8]. Therefore, it is included in the Thai National list of Herbal Products for use in the oral dosage form [9]. *Acmella paniculata* (Wall ex. DC.) R. K. Jansen (AP) (Syn. *Spilanthes paniculata* Wall ex. DC.) is a herb distributed throughout South Asia to Indochina Peninsula including Thailand and known to have a local anesthetic and anti-inflammatory activities; however, its toxicity has not yet been announced, and reports on the toxicity of this genus are very limited [10]. Although there are some reports regarding the efficacy of CQ in the treatment of hemorrhoids [11, 12], these reports used CQ in the oral dosage form and not combined with AP. Rectal suppositories containing combined standardized extracts of CQ and AP were formulated following the guideline of Thai Herbal Pharmacopoeia, and they were in acceptable quality based on physical and chemical properties. According to the Thai government policy to promote the use of herbal products in healthcare systems and goal 3 of sustainable development, ensure healthy lives and promote well-being for all at all ages, and we were interested in conducting a clinical pilot study to investigate the potential of rectal suppositories containing combined extracts of CQ and AP to alleviate the symptoms of hemorrhoids compared to a commercialized rectal suppository containing hydrocortisone and cinchocaine.

## 2. Materials and Methods

### 2.1. Study Design

This study was a multicenter, randomized, double-blind trial conducted in eight hospitals located in northern Thailand, namely, those in the Chiang Mai (*n* = 4), Lamphun (*n* = 1), Phayao (*n* = 1), Chiang Rai (*n* = 1), and Phitsanulok (*n* = 1) provinces, from May to June 2018. This study was conducted in accordance with good clinical practice and was approved by the Ethical Committee of the Faculty of Pharmacy, Chiang Mai University (study number 34/2017). The study project was funded by the Department of Thai Traditional and Alternative Medicine, Ministry of Public Health, and registered in the Thai Clinical Trials Registry (study ID: TCTR 20180501002).

### 2.2. Participants

Patients aged 18–70 years with grades I–III internal hemorrhoids confirmed by proctoscopy and either anal pain, bleeding, or both were enrolled in the study. All patients signed an informed consent form before screening. The exclusion criteria included a history of allergy to CQ and/or AP extract(s), pregnancy, breast feeding, menstrual period, history of anorectal surgery in the past year, malignancy, inflammatory bowel disease, immunocompromised status, anticoagulant use, and medication for hemorrhoids within 7 days prior to the initiation of the study. The discontinuation criteria were as follows: serious bleeding or serious adverse events considered unsafe by a physician, need to take corticosteroid in the oral or topical or injection form, missed follow-up at visit 2 or 3, and withdrawal from the study.

### 2.3. CQ-AP and the Commercialized Rectal Suppositories

CQ-AP suppositories containing CQ extract (1.0% w/w) and AP extract (10.0% w/w) were prepared in the Thai Traditional Medicine Laboratory of the Faculty of Pharmacy, Chiang Mai University, which has been certified for Good Manufacturing Practice. The resulting suppositories were administered to the CQ-AP group, while the commercialized rectal suppository containing hydrocortisone (5 mg) and cinchocaine (5 mg) was administered to the corresponding group.

### 2.4. Interventions

Patients were randomly assigned to receive either a CQ-AP (*n* = 53) or the commercialized rectal suppository ^®^ (*n* = 57). The patients were informed to take one suppository twice daily, in the morning, and at bedtime for 7 days. If defecation occurred within 30 minutes of application, another suppository would be reinserted. The patients in both groups were advised to consume a high-fiber diet and drink plenty of water without taking any laxatives. The patients were contacted via phone or home-visited by a nurse or practitioner of Thai traditional medicine on day 2, day 3, and day 7 to maximize adherence, monitor adverse drug events, and inform them of their next visit.

### 2.5. Outcomes

Patients were assessed on days 1, 4, and 8 by physicians for the following parameters: (1) primary outcome: bleeding severity (4-grade scoring system, 0–3) and prolapse size; (2) secondary outcome: anal pain using the visual analog scale (VAS) from 0 to 10.

### 2.6. Randomization, Blinding, and Allocation Concealment

Eligible patients (*n* = 110) were randomly allocated to two groups, CQ-AP and the commercialized rectal suppository, by a pharmacist in the participating hospitals, using a block randomization method with a block size of 4. The physicians, researchers, and statisticians were blinded to patient allocation. The patients were not informed about the type of suppository they received.

### 2.7. Statistical Methods

Statistical analysis was performed using repeated measures analysis of variance (ANOVA) for bleeding severity and anal pain in each group compared to baseline. When significance was found between each of the visits, the Bonferroni post hoc test was used for analysis. The analysis was subjected to intention-to-treat analysis, and the missing values were replaced using the last observation carried forward method. A *p* value of 0.05 or less was considered statistically significant.

## 3. Results

**Figure 1:**
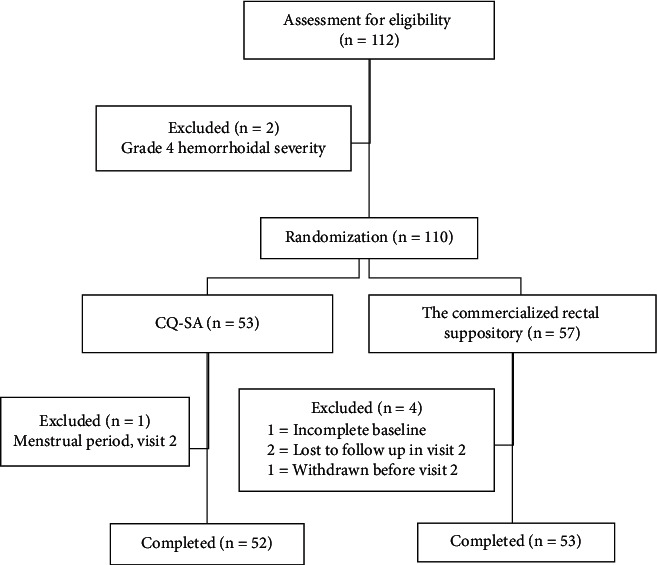
Schematic of patient selection.

Figure 1 shows the distribution and randomization of patients, including those in the primary analysis. Patients (*n* = 112) were screened for eligibility between May 2018 and June 2018, of whom seven were excluded. Two out of seven patients did not meet the inclusion criteria, whereas the reasons for excluding other patients were co-occurrence of the menstrual period (1), incomplete baseline data (1), request to withdraw (1), and missed follow-up (2). A total of 105 patients (52 in CQ-AP and 53 in the commercialized rectal suppository) completed the study.

Table 1 provides the demographic and baseline characteristics of the patients. There were no significant differences between the CQ-AP and the commercialized rectal suppository groups in terms of age, sex, presence of constipation, history of hemorrhoids, and duration of hemorrhoids. In each group, the majority were females with one prolapsed and grade 2 hemorrhoids.

Table 2 provides the clinical outcomes of the CQ-AP and the commercialized rectal suppository groups with respect to anal pain, bleeding, and prolapse on days 1, 4, and 8. Both treatments improved every clinical parameter and were comparable to each other. Bleeding, a primary outcome, was improved by over 80% and 90% on days 4 and 8, respectively, in both groups. The size of the prolapses was reduced by over 60% on day 8 in both groups. The CQ-AP group provided comparable anal pain relief to the commercialized rectal suppository group on day 8, eventhough greater pain relief was observed in the commercialized rectal suppository group on day 4.

Table 3 provides the satisfaction level of patients using the CQ-AP and the commercialized rectal suppositories, as well as their desire for further use of both treatments. Patients in both groups showed a high satisfaction level toward both treatments. The mean satisfaction score of the CQ-AP suppository was greater than that of the commercialized rectal suppository, although not statistically significant. Moreover, all patients showed a desire to use both suppositories in the future if hemorrhoids recurred.

Table 4 provides adverse events (AE) in patients using both treatments. In the CQ-AP group, there were 3 events, namely, anal pain, bleeding, and anxiety, found in 4 patients, accounting for 7.6%. In the commercialized rectal suppository group, ten patients experienced 12 events, accounting for 18.9%; anal pain and bleeding were more often found in patients using the commercialized rectal suppository compared to other events.

## 4. Discussion

This study revealed the potential effects of a rectal suppository containing CQ in combination with AP extracts. The therapeutic effects were found to be comparable to those of the commercialized rectal suppository, which contains hydrocortisone and cinchocaine, on hemorrhoids in a double-blind randomized controlled trial. This study was a multicenter study carried out in eight provinces in the northern part of Thailand, which contributes to the generalizability of the results.

Although there are reports regarding the efficacy of CQ in the treatment of hemorrhoids [11, 12], these were all administered orally and not in combination with AP. A study by Panpimanmas et al. [11] reported that the efficacy of CQ was comparable to that of micronized purified flavonoid fraction, as well as placebo, in terms of bleeding and pain. In another study, Lekutai and Pirshahid [12] showed that CQ and Cyclo 3 Fort^®^, which contains bioflavonoid hesperidin and an extract of *Ruscus aculeatus*, reduced bleeding, pain, and prolapse to a similar extent. Additionally, Kanket et al. demonstrated a comparable effect between orally administered CQ and flavonoids on bleeding, pain, and prolapse [13]. Another study used topical CQ extract applied three times a day compared to placebo and found that the effects of CQ extract on bleeding, pain, and prolapse were not significantly different from those of the placebo [14].

This study is the first to evaluate the efficacy of CQ extract in combination with AP extract as a rectal suppository, which is more likely to be well-tolerated compared to the oral dosage form. The efficacy of the CQ-AP and the commercialized rectal suppository for anal pain, bleeding, and prolapse on days 1, 4, and 8 were comparable. Bleeding, which was the primary outcome, was markedly improved in both groups. The size of the prolapses was markedly reduced by both treatments. The CQ-AP suppository provided comparable anal pain relief to the commercialized rectal suppository, although earlier onset of pain relief was observed in the commercialized rectal suppository group. The efficacy of CQ on anal pain, bleeding, and prolapse was in agreement with the findings of Panpimanmas and Lekutai [11, 12].

The effect of CQ on anal pain may be due to its anti-inflammatory activity [4, 5] and analgesic effect [6], whereas the local anesthetic effect and anti-inflammatory activity of AP [10] contribute to relieving pain, similar to hydrocortisone combined with lidocaine in the commercialized rectal suppository. Additionally, the cessation of bleeding and the reduction of prolapse size may be due to the vasoconstrictive effect of CQ [4].

In terms of tolerability, a few patients in the CQ-AP group experienced adverse events, including anal pain, bleeding, and anxiety. However, in the commercialized rectal suppository group, a greater number of patients experienced adverse events, namely, anal pain, bleeding, and mucus leakage from the anus. Some adverse events, such as anal pain and bleeding, were minor in severity and may not be related to the treatment, rather the signs and symptoms of hemorrhoids.

In addition to objective parameters, subjective parameters, such as patient satisfaction with both medications, were also assessed in this study. All recruited patients showed a high satisfaction with both treatments, and the mean satisfaction score of the CQ-AP suppository was insignificantly greater than that of the commercialized rectal suppository. Both groups were willing to use their corresponding treatments as needed.

The limitation of this study was the pilot design with no sample size calculation. Further studies should include a larger number of patients to obtain more information regarding efficacy and safety.

## 5. Conclusions

In conclusion, this pilot study demonstrated promising results regarding the potential efficacy of the combined extract of CQ and AP in rectal suppositories for hemorrhoidal treatment, with a good safety profile, as an alternative to conventional treatment, such as the commercialized rectal suppository and other suppositories containing similar active ingredients.

## Figures and Tables

**Table 1:** Patient demographics and characteristics.

Characteristics	CQ-AP	The commercialized rectal suppository	*P* value
	(*n* = 52)	(*n* = 53)	
Male (no. (%))	22 (42.3)	25 (47.2)	0.696
Age (years ± SD)	49.97 ± 12.76	52.77 ± 11.92	0.173
Presence of constipation (no. (%))	38 (73.1)	44 (83.0)	0.246
Duration of hemorrhoids (months) (median ± IQR)	30 ± 71	24 ± 71	0.465

Number of prolapse (no. (%))			0.870
0	0 (0.0)	1 (1.9)	
1	29 (55.8)	28 (52.9)	
2	12 (23.1)	15 (28.3)	
3	10 (19.2)	9 (17.0)	
4	1 (1.9)	0 (0.0)	

Grade of hemorrhoids (no. (%))			0.481
Grade 1	12 (23.1)	17 (32.1)	
Grade 2	25 (48.1)	20 (37.7)	
Grade 3	15 (28.8)	16 (30.2)	

**Table 2:** Clinical outcomes.

Outcomes		Mean ± SD		Median ± IQR		OR	95% CI	*P* value
		CQ-AP	The commercialized rectal suppository	CQ-AP	The commercialized rectal suppository			
Anal pain								
VAS (score)^*∗*^	Day 1	3.63 ± 2.39	3.17 ± 2.49	4.0 ± 3.0	3.0 ± 4.0	0.36	0.12–1.03	0.057
	Day 4	1.61 ± 1.74	0.82 ± 1.30	1.0 ± 2.0	0.0 ± 1.3			
	Day 8	0.80 ± 1.22	0.64 ± 1.26	0.0 ± 1.0	0.0 ± 1.0			

Bleeding								
Severity (score)^*∗∗*^	Day 1	1.23 ± 0.72	1.32 ± 0.78	1 ± 1	1 ± 1	1.47	0.34–6.32	0.610
	Day 4	0.21 ± 0.50	0.22 ± 0.64	0 ± 0	0 ± 0			
	Day 8	0.06 ± 0.31	0.16 ± 0.50	0 ± 0	0 ± 0			

Prolapse						Beta	95% CI	*P* value
Size (cm)	Day 1	1.31 ± 0.71	1.42 ± 0.72	1.0 ± 0.6	1.2 ± 0.9	−0.07	−0.23–0.10	0.414
	Day 8	0.76 ± 0.58	0.75 ± 0.53	0.5 ± 0.5	0.7 ± 0.6			
Size reduced (cm)^*∗∗∗*^		0.84 ± 0.68	0.87 ± 0.62	1.0 ± 1.2	1.0 ± 0.5			0.923

^*∗*^Pain severity measured by the visual analog scale ranging from 0 to 10, where 0 point indicates no pain at all and 10 points indicate the most severe pain. ^*∗∗*^Bleeding severity: 1 point for no bleeding, 2 points for having bleeding during some instances of defecation, 3 points for bleeding in every instance of defecation, and 4 points for large amount of bleeding during every instance of defecation. ^*∗∗∗*^Prolapse size reduction score: 0 point for insignificant size reduction, 1 point for 40–70% reduction, and 2 points for greater than 70% reduction. Data are expressed as the number (percentage) of patients or mean ± SD, unless otherwise indicated.

**Table 3:** Satisfaction level of patients.

	CQ-AP	The commercialized rectal suppository	*P* value
Satisfaction score (point)^*∗*^	3.31 ± 0.64	3.16 ± 0.78	0.288
Desire for further use (%)	52 (100.0)	53 (100.0)	1.000

^*∗*^Satisfaction score was classified as follows: 1 point for minimum satisfaction, 2 points for moderate satisfaction, 3 points for high satisfaction, and 4 points for maximum satisfaction. Data are expressed as the number (percentage) of patients or mean ± SD, unless otherwise indicated.

**Table 4:** Comparison of adverse events of the CQ-AP and the commercialized rectal suppository uses.

Events	CQ-AP	The commercialized rectal suppository
Anal pain	1 (1.9)	3 (5.7)
Bleeding	2 (3.8)	2 (3.8)
Anxiety	1 (1.9)	0 (0)
Burn feeling at the anus	0 (0)	1 (1.9)
Pelvic pain	0 (0)	1 (1.9)
Pain under the right breast	0 (0)	1 (1.9)
Anal pruritus	0 (0)	1 (1.9)
Oily droplet leak from the anus	0 (0)	1 (1.9)
White-yellowish mucus from the anus	0 (0)	1 (1.9)
Mucus leak from the anus	0 (0)	1 (1.9)

Data are expressed as the number (percentage) of patients unless otherwise indicated.

## Data Availability

The data used to support the findings of this study are available from the corresponding author upon request.
